# Chronic Subdural Hematoma Is Not Subdural: Anatomical, Biological, and Therapeutic Implications of a Misleading Definition

**DOI:** 10.3390/brainsci16060623

**Published:** 2026-06-10

**Authors:** Matteo De Simone, Elena Ciaglia, Alessandro Santurro, Anis Choucha, Benedetta Messuti, Stefano Fasolino, Rosario De Feo, Daniele Giuseppe Romano, Germano Guerra, Antonio De Luca, Giorgio Iaconetta

**Affiliations:** 1 Department of Medicine, Surgery and Dentistry “Scuola Medica Salernitana”, University of Salerno, Via S. Allende, 84081 Baronissi, Italy; eciaglia@unisa.it (E.C.); asanturro@unisa.it (A.S.); r.defeo9@stuednti.unisa.it (R.D.F.); daniele.romano@sangiovannieruggi.it (D.G.R.); giaconetta@unisa.it (G.I.); 2 Neurosurgery Unit, University Hospital “San Giovanni di Dio e Ruggi D’Aragona”, 84131 Salerno, Italy; 3 Legal Medicine Unit, University Hospital “San Giovanni di Dio e Ruggi D’Aragona”, 84131 Salerno, Italy; 4 Department of Neurosurgery, Aix Marseille University, APHM, UH Timone, 13005 Marseille, France; anis.c13@gmail.com; 5 Faculty of Medicine and Surgery TD, Department of Pharmacy, Health and Nutritional Sciences, University of Calabria, 87036 Rende, Italy; b.messuti@studenti.unisa.it; 6 Department of Biology and Chemistry “A. Zambelli”, University of Salerno, Via Giovanni Paolo II 132, 84084 Fisciano, Italy; 7 Department of Medicine and Surgery, Campus Bio-Medico University, Via Alvaro del Portillo 200, 00128 Roma, Italy; stefano.fasolino@alcampus.it; 8 Unit of Interventional Neuroradiology, University Hospital “San Giovanni di Dio e Ruggi D’Aragona”, 84131 Salerno, Italy; 9 Department of Medicine and Health Sciences “Vincenzo Tiberio”, University of Molise, Via F. De Santis, 86100 Campobasso, Italy; germano.guerra@unimol.it; 10 Department of Mental Health and Preventive Medicine, Section of Human Anatomy, University of Campania “L. Vanvitelli”, 81100 Naples, Italy; antonio.deluca@unicampania.it

**Keywords:** CSDH, MMA, craniotomy, DBH, intracranial hemorrhage

## Abstract

Chronic subdural hematoma (CSDH) is traditionally described as a post-traumatic blood collection within the subdural space; however, both its anatomical localization and pathophysiology have been increasingly questioned. Ultrastructural and histopathological evidence demonstrates that no true subdural space exists under physiological conditions and that CSDH originates instead within the dural border cell (DBC) layer, a mechanically fragile and biologically active meningeal interface. Accordingly, chronic “subdural” hematoma may be more accurately interpreted as an intradural border cell lesion. Beyond anatomy, CSDH is a dynamic, self-sustaining disease driven by chronic inflammation, pathological angiogenesis, vascular immaturity, and localized hemostatic dysregulation. Hypoxia-induced HIF-1α/VEGF activation promotes fragile, hyperpermeable neovessels, while local hyperfibrinolysis and kallikrein–kinin activation prevent stable clot formation, driving recurrent microbleeding and plasma exudation. Consequently, hematoma persistence and recurrence represent a biological failure rather than a purely technical surgical shortcoming. This conceptual shift provides a coherent rationale for dural-targeted therapies, including middle meningeal artery embolization and pharmacological modulation of angiogenesis and fibrinolysis. Reframing CSDH as a chronic intradural and biologically active disorder has important implications for terminology, classification, and the development of mechanism-oriented, multidisciplinary management strategies.

## 1. Introduction: Why Chronic Subdural Hematoma Is No Longer a “Simple” Disease

Chronic subdural hematoma (CSDH) has long been regarded as a post-traumatic collection of blood within the “subdural space” [1]. However, what has been recognized for many years is that this condition is not invariably post-traumatic [2], and that this “subdural space” does not exist [3]. These two considerations represent the keystone for the whole paper, which will be taken up and deepened in the following sections. Two aspects are increasingly recognized in relation to this condition: its rising epidemiological burden and the ongoing development of new treatment strategies. In a contemporary population-based study from a representative United States region, the incidence of CSDH was estimated at 16.3 cases per 100,000 persons per year, corresponding to an age-, sex-, and race-standardized national estimate of 17.3 cases per 100,000 persons per year. Incidence increased markedly with age and was higher in men, reaching 354.8 cases per 100,000 persons per year [95% CI 242.7–500.9] among men aged 85 years or older [4]. The observed increase in incidence results from a combination of factors. Its impact is considerable, not only in medical terms but also economically, since costs related to the management of CSDH are projected to rise and should be anticipated within healthcare systems. Two additional features further contribute to the complexity of dealing with this condition: approximately 25% of patients present with CSDH of unknown etiology, and recurrence occurs in 2.3% to 33% of cases, depending on patient- and treatment-related factors. Among patients who experience recurrence, three main risk factors—male sex, bilateral hematoma, and the absence of postoperative drainage—have been identified with convincing evidence [5]. Regarding management, while middle meningeal artery embolization represents an innovative and potentially effective strategy, there is still insufficient evidence to determine whether it is best employed as a primary treatment or as an adjunct to surgery for recurrence prevention [6]. In addition, its economic sustainability has yet to be fully evaluated [7].

Taken together, these epidemiological trends, biological uncertainties, and high recurrence rates support the view that CSDH should not be regarded solely as a simple, static, or purely mechanical entity. Rather, CSDH should be understood as a dynamic and biologically active disease, shaped by complex interactions between local vascular pathology, inflammation, coagulation imbalance, and treatment-related factors. In this paper, we aim to reframe CSDH through this modern lens, integrating emerging pathophysiological evidence to move beyond traditional paradigms and toward a more mechanism-driven understanding of its persistence, progression, and recurrence.

## 2. From “Subdural” to “Intradural”: Rethinking the Anatomical Definition

Although the “subdural space” was historically described as a mesothelial-lined, fluid-containing cavity between the dura and the arachnoid, contemporary ultrastructural evidence indicates that no true (actual or potential) space exists at this junction in the intact state [8].

In the classic anatomical description, the dura mater is recognized as having a histological stratification into three layers; from superficial to deep, there are periosteal, meningeal, and border zone layers [9,10]. It is in the latter that a specific group of cells, the dural border cells, can be identified, well described and studied by Dr. Haines in his seminal study [3].

At the dura–arachnoid junction, the dural border cell (DBC) layer represents the innermost portion of the dura mater and forms a structurally distinct, mechanically vulnerable interface with the arachnoid barrier cell layer. Ultrastructurally, DBCs are flattened, modified fibroblasts with long, tortuous and interdigitating processes, few cell junctions (predominantly desmosomes with occasional gap/intermediate junctions) [11], no organized extracellular collagen or elastic fiber framework, and irregular extracellular clefts containing amorphous non-fibrillar material [12]. Furthermore, the meningeal dura mater is richly supplied by branches of the middle meningeal artery (MMA), whereas the arachnoid barrier cell layer is essentially avascular and structurally reinforced by numerous tight junctions. Interposed between these compartments, the dural border cell (DBC) layer represents a mechanically vulnerable, normally avascular plane that lies in close proximity to the inner dural microcirculation. Under pathological conditions, this layer undergoes cleavage and remodeling, becoming infiltrated by fragile neocapillaries derived from dural vessels, particularly the MMA. Histopathological studies consistently demonstrate that the vascularized outer membrane of chronic subdural hematoma is dural in origin [13], while the inner membrane is relatively avascular and composed predominantly of cells morphologically consistent with dural border cells [14].

Within this anatomical framework, the DBC layer constitutes a structural plane of weakness susceptible to cleavage by mechanical or traumatic shearing forces. Rather than the expansion of a pre-existing “potential” cavity, this cleavage arises from true tissue disruption, marked by widened extracellular spaces, ruptured intercellular junctions, and cellular membrane injury. Whilst experimental infusion studies consistently demonstrate that blood preferentially dissects the DBC layer, pathological specimens reveal DBC-like elements distributed across both the outer dural and inner arachnoid-facing components of the CSDH capsule. These observations strongly support the paradigm that chronic “subdural” collections are more accurately conceptualized as intra-dural lesions rather than accumulations within a true subdural space [15].

Building on this anatomical and pathological substrate, the clinical observations reported by Shapiro et al. [9] provide a relevant functional correlation. Although their study does not directly address the ultrastructural anatomy of the dura–arachnoid interface, the therapeutic efficacy of middle meningeal artery embolization suggests that the biological mechanisms sustaining hematoma persistence and recurrence are predominantly dural-based, arising from the pathological neovascularization of the hematoma membranes supplied by the middle meningeal artery. By selectively interrupting this vascular input, embolization appears to disrupt the cycle of microhemorrhage, inflammation, and membrane maintenance, a mechanism anatomically consistent with lesion development within the dural border cell layer rather than within a pre-existing subdural cavity.

Accordingly, several authors have argued that “subdural hematoma” is often a misnomer [16,17] and that the term “dural border hematoma” (DBH) better localizes the lesion to a specific meningeal compartment. Ultimately, this anatomical reappraisal reinforces the classical principle that *nomina sunt consequentia rerum*: terminology must follow biological reality. Reconsidering chronic ‘subdural’ hematoma as a DBH may represent more than a semantic exercise but reflects a more accurate understanding of its origin and behavior.

In this perspective, reappraising chronic “subdural” hematoma through the lens of meningeal microanatomy shifts the focus from a hypothetical anatomical space to a specific, structurally vulnerable meningeal layer. Integrating ultrastructural, pathological, and therapeutic evidence highlights the DBC layer as the true substrate of disease. Such a framework not only clarifies long-standing anatomical ambiguities but also provides a rational basis for emerging dural-targeted treatments.

## 3. Beyond Trauma: A New Pathophysiological Paradigm

As early as the mid-20th century, it was recognized that not all chronic subdural hematomas arise from overt traumatic events. In a landmark observation, Rowbotham and Little [2] distinguished a subgroup of patients in whom no history of trauma could be identified and in whom surgical drainage alone yielded disappointing outcomes. They proposed that, in these cases, the hematoma originates from repeated low-grade bleeding of the dural microvasculature rather than from rupture of bridging veins. Crucially, they argued that the dominant pathological process was not mechanical compression, but progressive vascular degeneration within the intracranial environment. This early insight anticipated the modern view of CSDH as a disease driven by ongoing biological dysfunction rather than a single traumatic insult, laying the foundation for a paradigm that extends beyond trauma and reframes chronic subdural hematoma as a dynamic, self-perpetuating pathological process.

Building on these early observations, contemporary research has increasingly shifted the understanding of chronic subdural hematoma toward a biologically driven disease model [18,19,20,21].

From an inflammatory standpoint, patients with CSDH exhibit elevated concentrations of the pro-inflammatory cytokines IL-6 and IL-8 in both subdural and peripheral compartments [22,23,24]. This observation is complemented by evidence indicating that lower levels of the anti-inflammatory cytokine IL-10 across these compartments are associated with a reduced risk of recurrence, whereas increased concentrations of interleukin-1 receptor antagonist (IL-1ra) are linked to higher recurrence rates [25]. In parallel, markedly raised levels of vascular endothelial growth factor (VEGF) and its receptor within the subdural fluid, together with increased fibrin/fibrinogen degradation products, reflect a concomitant dysregulation of angiogenic and haemostatic pathways [21,26]. At the cellular level, several studies have examined peripheral blood parameters in relation to clinical outcomes, demonstrating that systemic factors such as malnutrition and elevated neutrophil counts are significantly associated with an increased likelihood of recurrence [27].

Collectively, these alterations provide a physiopathological explanation for ongoing micro-haemorrhage and the propensity for hematoma formation, persistence and recurrence.

In the subsequent sections, each of these three interrelated mechanisms of dysregulation—inflammation, haemostasis, and neoangiogenesis—is examined separately and in a detailed, mechanism-specific manner.

## 4. Immune Profiling in CSDH

Jensen et al. [27] first demonstrated that the subdural fluid from CSDH patients contains immune cells, RBC, and thrombocytes, and that the subdural cellular composition of granulocytes, lymphocytes and monocytes is significantly different from a reference of normal blood. In patients with recurrent CSDH, the subdural cellular composition differed between the first and second operation, though no such difference was found at primary surgery between patients who later developed recurrence and those who did not. Notably, subdural mean corpuscular volume (MCV) at primary surgery was significantly higher in patients who subsequently developed recurrent CSDH, emerging as a potential early predictive marker of recurrence. MCV is a measure of the average volume of RBC and MCV is typically an indicator of anemia, but in cases of macrocytosis, MCV may serve as a biomarker of inflammation and endothelial function [28,29], pointing toward pathophysiological inflammatory involvement on a cellular level. Later, a pilot study by Ciaglia et al. [20] employing multiparametric flow cytometry characterized for the first time the immune compartment of subdural fluid in detail, demonstrating that it is dominated by innate cytotoxic cells—CD8^+^ T lymphocytes, NK and NKT cells—with a composition significantly distinct from peripheral blood, potentially regulated by the CX3CR1/fractalkine axis. Among the key novel findings is the first report of a massive NKT lymphocyte presence in subdural fluid, cells with a controversial role in cerebral homeostasis [30], whose local expansion suggests a pathological involvement in brain injury. Equally relevant is the identification of an erythro-myeloid progenitor population of CD31+CD45+ cells, absent in healthy donor blood and significantly correlated with circulating platelets in patients, which emerges as a potential peripheral biomarker for ongoing central hematoma activity. The cytokine profile—with elevated IL-6 and IL-8 in the subdural compartment and reduced TNF-α compared to peripheral blood—points to a local brain-derived source driving chemotactic migration of endothelial progenitors. Overall, monitoring these cellular populations may represent a non-invasive auxiliary diagnostic tool, opening future perspectives for targeted immunotherapeutic approaches and larger-scale studies.

## 5. Neoangiogenesis and Vascular Fragility

The progression of CSDH is no longer viewed as a static mechanical accumulation of blood, but rather as a dynamic, auto-perpetuating angiogenic and inflammatory disease [31,32,33]. Central to this paradigm shift is the formation of a highly vascularized neomembrane, primarily on the dural surface, which serves as the biological engine for hematoma expansion and recurrence. Unlike physiological wound healing, which transitions from an initial sprouting to vessel stabilization followed by a regression, the CSDH microenvironment is characterized by a locked “early granulation” phase where neoangiogenesis fails to reach full maturity.

### 5.1. Molecular Drivers of Chronic Angiogenetic Activation

The sustained angiogenic drive within the CSDH neomembrane is orchestrated by a complex network of growth factors, with VEGF acting as the central driver [34]. Quantitative analyses reveal that VEGF concentrations in hematoma fluid are significantly higher than in systemic serum, established by local production from infiltrating inflammatory cells, particularly macrophages [35].

This pathological overexpression is fundamentally triggered by local hypoxia within the hematoma cavity. Low oxygen tension activates Hypoxia-Inducible Factor-1α (HIF-1α), which directly upregulates VEGF transcription [36]. This HIF-1α/VEGF axis promotes a cycle of abnormal vessel formation and increased permeability via multiple intracellular cascades:

(1) PI3K/Akt Pathway: Regulates endothelial cell survival, proliferation, and vascular permeability.
(2) NF-κB Activation: Promotes a pro-inflammatory microenvironment that sustains pathological angiogenesis.
(3) Synergistic Growth Factors: Factors such as basic fibroblast growth factor (bFGF) and placental growth factor (PlGF) further amplify this pro-angiogenic milieu/niche [37].

### 5.2. Structural Immaturity and the Angiopoietin/Tie2 Imbalance

Neoangiogenesis in CSDH is distinct from physiological repair due to a critical imbalance in vessel maturation signals. While VEGF initiates sprouting, vessel stabilization normally requires the coordinated action of the Angiopoietin (Ang)/Tie2 system and Platelet-Derived Growth Factor (PDGF). In CSDH neomembranes, according to literature, there is a pathologically high Ang2/Ang1 ratio. Elevated Ang2 acts as a context-dependent antagonist, destabilizing existing vessels and preventing maturation [31,33].

This molecular environment results in neovessels characterized by profound structural immaturity. Histopathological and ultrastructural evidence demonstrates [31,33]:

- Endothelial Junctional Defects: Fragile capillaries exhibit widened intercellular gap junctions and fenestrations [38,39,40].
- Basement Membrane Deficiency: Vessels often possess incomplete or entirely absent basement membranes.
- Pericyte Coverage Failure: There is a notable lack of pericyte recruitment and structural support, largely attributed to deficient levels of PDGF-BB and Transforming Growth Factor-β1 (TGF-β1).

**Figure 1:**
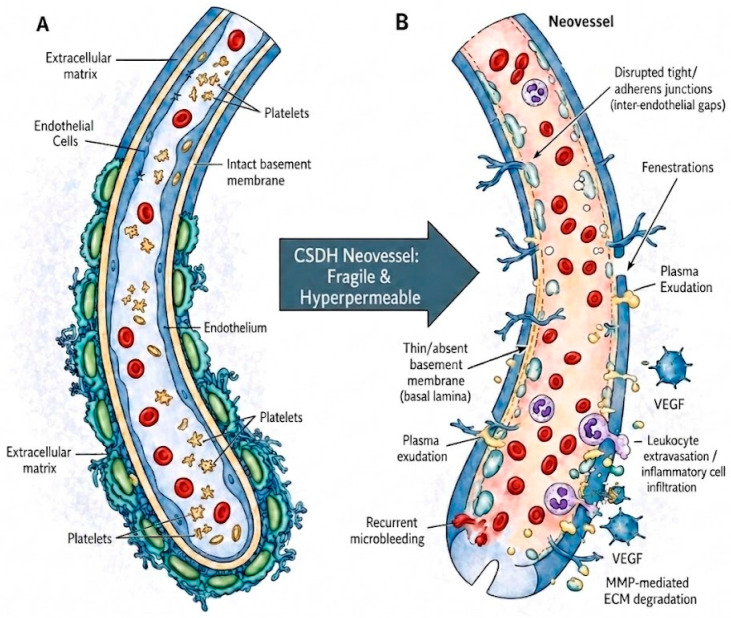
Conceptual model of vascular maturation versus pathological neovessels in chronic subdural hematoma (CSDH). (**A**) Mature microvessel with intact endothelial junctions, continuous basement membrane, and organized pericyte coverage supporting barrier integrity. (**B**) CSDH-associated neovessel characterized by disrupted tight junctions with inter-endothelial gaps, fenestrations, discontinuous basal lamina, reduced mural support, and matrix remodeling (MMP-mediated degradation). These alterations promote plasma exudation, leukocyte extravasation/inflammatory infiltration, and recurrent microbleeding, consistent with VEGF-driven pathological angiogenesis sustaining hematoma persistence.

These “giant” sinusoidal capillaries are inherently hyperpermeable, facilitating the continuous transendothelial filtration of plasma proteins and the extravasation of erythrocytes into the hematoma cavity. These features collectively define a fragile, hyperpermeable microvascular bed (Figure 1).

Importantly, the hyperpermeability of CSDH-associated neovessels should not be interpreted as classical brain parenchymal blood–brain barrier (BBB) disruption. The classical BBB is formed by specialized parenchymal endothelial cells supported by tight junctions, pericytes, basement membrane components, and astrocytic end-feet [41,42]. By contrast, the leaky vessels described in CSDH arise predominantly within the dural border cell/neomembrane compartment, in close relationship with the middle meningeal artery vascular territory, rather than within cortical parenchymal microvessels [31,33,39]. These vessels are structurally immature, with thin or discontinuous basement membranes, deficient mural-cell or pericyte support, endothelial junctional defects, and inter-endothelial gaps, features that may facilitate plasma exudation, inflammatory cell trafficking, and recurrent microbleeding [31,33]. In this respect, CSDH shares several mechanisms with other angiogenesis-driven disorders, including tumor angiogenesis and diabetic retinopathy, in which VEGF excess, Ang-2–mediated vascular destabilization, matrix metalloproteinase activity, basement membrane remodeling, and impaired pericyte-endothelial stabilization contribute to immature and hyperpermeable neovessels [43,44]. However, this analogy is mechanistic rather than anatomical: CSDH-associated leakiness reflects pathological meningeal angiogenesis within the dural/neomembrane compartment, not primary breakdown of the parenchymal neurovascular-unit BBB. Other CNS interfaces, including the arachnoid barrier, blood–CSF interfaces, and meningeal lymphatic pathways, may further influence local fluid exchange, inflammatory mediator clearance, and immune-cell compartmentalization [39,45,46]. Thus, CSDH may be more appropriately considered a compartmentalized meningeal barrier and wound-healing disorder, in which dural border cell-layer remodeling, VEGF-driven neovascularization, recurrent microbleeding, and impaired clearance mechanisms cooperate to sustain the hematoma membrane [31,33].

### 5.3. The Vicious Cycle of Fragility and Recurrent Microbleeding

CSDH expansion is sustained by fragile outer membrane neovessels prone to rupture, a process exacerbated by MMP-2- and MMP-9-mediated degradation of the vascular basement membrane and extracellular matrix [47].

Recurrent microbleeding sustains a vicious cycle in which hemoglobin degradation products activate macrophages, leading to cytokine release and further VEGF- and MMP-mediated inflammation and angiogenesis [48]. This mechanism helps explain why surgical evacuation alone may fail: although the hematoma is removed, the pathological neomembrane persists, and its fragile, leaky neovessels continue to exude fluid and blood [33].

### 5.4. Therapeutic Implications of Vascular Targeting

The recognition of CSDH as a primarily angiogenic disease provides the biological rationale for non-surgical interventions. Corticosteroids such as dexamethasone reduce recurrence by suppressing VEGF expression and stabilizing the neomembrane vasculature, although systemic morbidity limits their utility [49]. Targeted anti-angiogenic agents, such as bevacizumab, have shown experimental efficacy in reducing hematoma volume by blocking the VEGF-mediated permeability cascade [50]. Furthermore, MMA embolization targets this pathological network by cutting off the arterial supply to these fragile capillaries, supporting the relevance of neovascularization in disease persistence [51].

## 6. Hemostatic Dysregulation and Recurrent Microbleeding

Although fragile neovascularization provides the structural basis for CSDH, its persistence and expansion are driven by profound dysregulation of the local biochemical environment [31,33,52]. Available evidence supports the view that the hematoma cavity does not act as a simple collection space, but rather behaves as a biologically active compartment characterized by profound dysregulation of hemostasis, a vicious cycle of inflammation, and widespread fibrosis [53,54]. This may lead to a network of immature vessels embedded in a hypertrophic fibrotic stroma. Chronic hematomas have long been known to persist in a liquid state due to elevated local fibrinolysis. A 2006 Journal of Neurosurgery study showed higher tissue plasminogen activator levels in lesions than in serum, and significantly increased TPA levels in recurrent compared with non-recurrent hematomas [55]. Elevated tissue plasminogen activator (TPA) levels indicate incoagulable blood, as TPA converts plasminogen into plasmin—a serine protease that degrades fibrin and dissolves clots. Increased TPA in recurrent cases highlights its role in postoperative recurrence and its utility as a predictor of lesion instability. Consequently, research has focused on elucidating these molecular mechanisms and identifying staging markers for CSDH.

Along these lines, Park et al. (2011) [56] categorized 31 CSDH patients based on CT internal architecture into heterogeneous (layering/mixed density) and homogeneous (high/iso/low density) groups. Subsequent analysis revealed significantly higher D-dimer levels—a fibrin degradation product indicating hyperfibrinolysis—in the heterogeneous group (35,407.9 ± 16,325.5 μg/L) compared to the homogeneous group (1476.4 ± 2091.4 μg/L), both far exceeding the normal healthy threshold of less than 500 μg/L.

These findings demonstrate that fibrinolytic activity is massive during the heterogeneous phase and milder during the homogeneous phase. Pathophysiologically, acute post-hemorrhagic clot formation triggers a fibrinolytic response that inadvertently compromises vascular integrity, driving recurrent microhemorrhages. This mixture of old dissolved blood and fresh bleeding creates the “heterogeneous” CT appearance. As the cycle slows, the blood liquefies into a uniform fluid, entering the “homogeneous” phase—a deceleration directly supported by the observed decline in D-dimer levels [56].

New findings regarding these mechanisms have drawn attention to potential therapeutic implications. In addition to TPA, urokinase (UPA) has also been shown to be involved in this homeostatic dysregulation, which prevents the formation of a stable clot [57].

High levels of UPA have been found in hematoma fluid. A significant role of the hematoma’s outer membranes in the production of TPA has also been demonstrated, thus identifying the hematoma itself, with its membranes, as the active source of hyperfibrinolysis [31,57].

**Figure 2:**
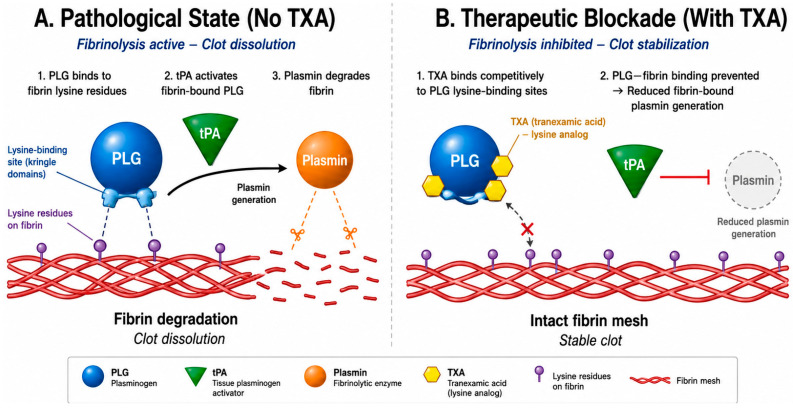
Molecular mechanism of tranexamic acid (TXA). (**A**) In the absence of TXA, plasminogen (PLG) binds to lysine residues exposed on the fibrin mesh through its lysine-binding sites. Tissue plasminogen activator (tPA) then promotes the conversion of fibrin-bound PLG into plasmin. Locally generated plasmin degrades fibrin, contributing to fibrin mesh breakdown and clot dissolution. (**B**) In the presence of TXA, TXA molecules, shown as yellow hexagons, act as synthetic lysine analogs and bind competitively to the lysine-binding sites of PLG. This reduces PLG binding to fibrin, limits local plasmin generation, and helps preserve the fibrin mesh, thereby contributing to clot stabilization.

tPA, together with inflammation and vascular hyperpermeability, may contribute to a self-perpetuating cycle that promotes hematoma expansion. These observations have stimulated interest in pharmacological strategies aimed at modulating local fibrinolysis. Tranexamic acid (TXA) is an antifibrinolytic agent and synthetic lysine analog that reduces fibrinolysis by reversibly binding to lysine-binding sites on plasminogen and plasmin. By occupying these sites, TXA limits the interaction between plasminogen and fibrin, thereby reducing fibrin-associated plasmin generation and subsequent fibrin degradation. In this way, TXA may help stabilize the fibrin clot within the hematoma cavity, as illustrated schematically in Figure 2.

Fujisawa et al. [58] demonstrated that hyperfibrinolysis alone does not fully account for CSDH pathology, identifying increased vascular permeability via kallikrein–kinin system activation as a crucial secondary mechanism. This system involves the consumption of two inactive precursors, prekallikrein and high-molecular-weight (HMW) kininogen, to produce bradykinin—a potent mediator of vasodilation and endothelial permeability.

In a study of 119 patients, the authors found significantly lower levels of prekallikrein and HMW kininogen in hematoma fluid compared to peripheral blood, alongside significantly elevated bradykinin levels. This consumption profile confirms local kallikrein–kinin activation. The resulting high concentration of plasma proteins within the hematoma further underscores this bradykinin-driven capillary hyperpermeability and subsequent plasma exudation. Corroborating these findings, microscopic examination of the outer hematoma membrane revealed edema, perivascular hemorrhage, and leukocyte migration, providing direct histological evidence of a leaky endothelial barrier [58].

In contrast to healthy vessels where tightly packed endothelial cells fully shield the underlying basement membrane, the capillaries of CSDHs exhibit interendothelial gaps. Upon endothelial disruption or microbleeding, blood and hematoma fluid come into direct contact with the negatively charged components of the exposed basement membrane, such as collagen. This exposure triggers the intrinsic pathway: circulating Factor XII (FXII) autoactivates upon surface contact, subsequently activating the FXI-FIX-FVIII cascade to convert Factor X, ultimately driving fibrin clot formation to arrest bleeding.

However, local hyperfibrinolysis dismantles this fibrin plug, releasing plasmin. Rather than acting solely on fibrin, plasmin cleaves circulating, unbound FXII, removing its surface-binding domain. The resulting soluble FXII fragment detaches into the hematoma fluid, losing its procoagulant ability to form subsequent clots. Instead, this fragment transforms into a potent fluid-phase activator of the kallikrein–kinin system, driving the pathological production of bradykinin.

This action of plasmin isn’t unique to hematomas and has been well described by Jukema et al. [59]. Since it is detached, this fragment—known as the FXIIa β-chain—can no longer effectively activate coagulation, a process that requires a surface. Instead, it becomes highly efficient at converting prekallikrein into kallikrein. This protease, in turn, cleaves HMW kininogen, leading to the release of bradykinin. Bradykinin maintains blood vessels in a state of chronic leakiness, promoting fluid accumulation and membrane edema, thereby sustaining the persistence of the pathology itself. The central role of this mechanism in generating the chronic leakiness characteristic of the hematoma has recently been confirmed by Arunachalam and colleagues [54].

In the continuous and unsuccessful attempt to treat microbleeding from neovascularization, platelets and coagulation factors are recruited and depleted in situ. The net result is a blood fluid depleted of functional coagulation factors, rich in fibrin degradation products (such as the previously mentioned D-dimer) and degranulated platelets that, by releasing their contents (including VEGF and PDGF), paradoxically end up fueling inflammation rather than resolving the hemorrhage.

Acquired coagulopathies can aggravate the situation. A recent case report published by Otsuka et al. describes the case of an 82-year-old patient with a chronic subdural hematoma and a rare, acquired coagulopathy (deficiency VII, XI, XII). This is clearly a patient at high risk for bleeding, for whom traditional surgery is ineffective or dangerous. The case was definitively resolved with embolization of the MMA using cyanoacrylate glue; this endovascular technique occludes the vessels feeding the hematoma membrane [60].

**Figure 3:**
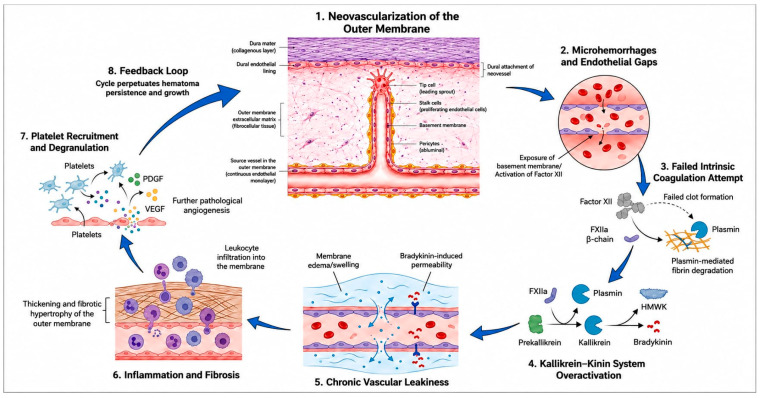
Schematic overview of the self-perpetuating pathophysiological mechanisms underlying CSDH. (1) Pathological neovascularization of the outer membrane leads to the formation of fragile, immature microvessels. (2) Endothelial gaps and microhemorrhages promote blood extravasation. (3) Intrinsic coagulation is inadequately activated, resulting in ineffective clot formation. (4) Overactivation of the kallikrein–kinin system enhances fibrinolysis and vascular permeability. (5) Persistent vascular leakiness sustains fluid accumulation. (6) Chronic inflammation and fibrosis drive membrane thickening. (7) Platelet recruitment and degranulation release pro-angiogenic mediators. (8) A positive feedback loop maintains angiogenesis, inflammation, and recurrent bleeding, promoting hematoma persistence and growth.

As for the platelets present in the hematoma, we said that they release large amounts of PDGF (and other inflammatory substances). PDGF binds to pericytes (cells that surround and support blood capillaries) and signals them to activate and build new blood vessels in a process of angiogenesis. However, the vessels generated in this circumstance are often immature, fragile, and leaky. This inevitably causes a vicious cycle. These new vessels, in fact, are weak and tend to break easily, causing continuous microhemorrhages within the hematoma cavity. Our body, in reaction to these microhemorrhages, will recruit new platelets, which release more PDGF, and the situation returns to the starting point (Figure 3).

A study published by Yokota et al. in 2024 [61], investigating the reasons why CSDH tends to grow or not heal spontaneously, demonstrates that precisely this role of PDGF-B in activating pericytes to continuously form blood microvessels in the membrane of the hematoma promotes healing, while the chronic environment of CSDH leads to pathological fibrosis. Profibrotic cytokines, such as TGF-beta, cause the deposition of a thick collagen matrix in the outer membrane. This fibrotic remodelling may worsen CSDH for two main reasons:

(1) First, the formation of a rigid capsule prevents brain re-expansion after drainage, leaving a dead space that facilitates recurrence. To clarify: the dead space is the space left between the brain and the skull after drainage. If the membrane is rigid, the brain is unable to re-expand to fill that void. The empty space therefore tends to refill with fluid or blood.
(2) Second, the immature new vessels remain trapped in the rigid fibrotic stroma, which prevents their natural mechanical vasoconstriction. The vessels remain open, perpetuating microbleeding.

This fibrosis mechanism has been well described by Edlmann et al. [31], who define fibrosis as a failed repair attempt. Trauma separates the cells of the dural border, triggering a response similar to skin wound healing: a so-called “fibroproliferation.” However, instead of closing the wound and stopping it, the process goes out of control: collagen synthesis outpaces its degradation, creating increasingly thicker membranes. The very high levels of collagen precursors (procollagen type 1 and type 3) found in the hematoma fluid (much higher than in peripheral blood) indicate the presence of an ongoing fibrotic process. Eosinophils release TGF-β1, which activates an intracellular signaling pathway called the SMAD pathway. This signal directs persistent fibrosis and membrane formation (Table 1).

## 7. Management

Despite its high prevalence, CSDH management remains a matter of ongoing debate, both regarding surgical indications and therapeutic modalities [62]. For decompensating patients presenting with profound neurological symptoms, emergent surgical drainage is the standard of care. However, there is a paucity of evidence to guide treatment decisions for less threatening situations, making clinical judgment a key element in the decision process [63]. Treatment decisions integrate clinical factors (impaired consciousness, neurological deficits, seizures), radiological features (hematoma thickness, mass effect, midline shift, Nakaguchi morphology [64], location, etc.), and patient-specific considerations such as age, antithrombotic therapy and other risk factors of poor postoperative outcome. The following subsections address conservative management, surgical evacuation, and middle meningeal artery embolization.

### 7.1. Conservative Management

For patients with mild to absent symptoms, particularly those at high operative risk, conservative management is often the initial strategy. Without surgery, approximately 40% of cSDHs spontaneously resolve, though around 20% of conservatively managed patients ultimately require intervention [65]. To this day, clinical practice remains largely non-standardized.

Oral hydration (2 L/day) is commonly recommended as a safe supportive measure, though evidence remains limited [65]. Regarding antithrombotic therapy, discontinuation with or without reversal, is generally considered in symptomatic patients, with resumption timing decided on a patient-specific basis [63].

Among pharmacological options, atorvastatin demonstrated significantly greater hematoma volume reduction, better neurological outcomes, and lower surgical bailout rates in an RCT of 196 patients by Jiang et al. [66]. Tranexamic acid has also shown promise: a meta-analysis by Musmar et al. (*n* = 1379) reported significantly lower recurrence (OR 0.35; *p* < 0.01) and reduced 3-month hematoma volume (Δ − 4.56 mL; *p* = 0.03) without increased complications [67]. Corticosteroids remain controversial: two RCTs, Hutchinson et al. (*n* = 748) [68] and Miah et al. (*n* = 252) [69], both reported unfavorable outcomes with dexamethasone compared to placebo or surgery. However, low-dose hydrocortisone has shown more promising results in smaller series, though data remain underpowered [70]. Bevacizumab represents a novel but still experimental option for refractory cases [71].

Follow-up imaging intervals remain undefined and are tailored to each patient’s clinical specificity and hematoma characteristics.

A key limitation of this literature is that most trials compare medical therapies against surgery rather than placebo, making it difficult to assess the true efficacy of conservative treatment in isolation. Rates of spontaneous resolution remain poorly defined, and available evidence yields mixed results overall.

### 7.2. Surgical Evacuation

Surgical evacuation is generally indicated in patients with significant neurological symptoms, typically associated with hematoma thickness >10 mm or midline shift >5 mm. However, no formal evidence-based algorithm exists to guide these decisions, and surgical indications remain largely based on clinical judgment and individual case assessment. Surgery carries non-negligible risks, with morbidity and mortality ranging 3.0–56.8% and 2.7–30%, respectively, and recurrence rates reaching up to 39% [72].

Three main techniques are employed: burr hole craniostomy or minicraniotomy with irrigation and drainage (the most widely used) [73], twist drill trephination (a minimally invasive bedside approach well-suited to elderly or high-risk patients) [74], and large craniotomy (reserved for complex, organized, or recurrent hematomas). Endoscopy-assisted evacuation has shown promising low recurrence rates in limited series but lacks standardization [75]. Across techniques, a large meta-analysis of 34,829 patients found no significant difference in mortality, cure, or recurrence between burr hole irrigation and twist drill trephination [76].

The use of a postoperative closed-system drain stands as the most robustly supported technical factor. In a network meta-analysis of 103,645 cases, Henry et al. demonstrated that drain placement significantly reduces recurrence (iRR 0.53, 95% CI 0.44–0.63) without increasing morbidity (iRR 0.97) [77]. Notably, drain position (subdural versus subgaleal) does not influence recurrence outcomes [1]. Drain duration of 24 or 48 h appears equivalent in terms of recurrence; however, 6 or 12 h are associated with more complications [78].

Several other technical aspects remain subject to debate. The number of burr holes (one versus two) has not been shown to significantly impact recurrence or complication rates. Subdural irrigation reduces reoperation rates; however, the risk of pneumocephalus or cortical injury is not negligible [79], with no consensus on optimal irrigation volume (protocols vary between 200 and 500 mL per side). Finally, the choice of anesthesia (local versus general) does not influence recurrence [80], and local anesthesia is generally preferred in elderly patients for its favorable safety profile.

### 7.3. Middle Meningeal Artery Embolization

Middle meningeal artery embolization (MMAE) targets the pathological vascularization of subdural neomembranes, breaking the self-perpetuating cycle of hemorrhage and inflammation. The procedure is endovascular and minimally invasive, performed via selective catheterization of the MMA. Several embolic agents are available, including liquid embolics (Onyx, Squid, n-BCA), polyvinyl alcohol (PVA) particles, and coils. In a systematic review and meta-analysis of 22 studies, Ku et al. [81] reported that Onyx demonstrated the lowest rates of recurrence (2%), reoperation (2%), and complications (2%), while PVA combined with coils yielded the highest rate of good clinical outcomes (85.2%). Across all agents, overall recurrence, reoperation, and complication rates were 4.1%, 4.2%, and 2.6%, respectively.

The scientific evidence for adjunctive MMAE has been recently strengthened by two RCTs. The EMBOLISE trial (Davies et al., NEJM 2024) [82] randomized 400 patients with symptomatic CSDH requiring surgery to adjunctive MMAE with Onyx versus surgery alone; reoperation occurred in 4.1% versus 11.3% (RR 0.36; 95% CI 0.11–0.80; *p* = 0.008), with procedural serious adverse events in 2.0%, including two disabling strokes. The STEM trial (Fiorella et al., NEJM 2025) [83] randomized 310 patients with symptomatic cSDH into MMA embolization as an adjunct to standard treatment versus standard treatment alone. Standard treatment could be surgery or conservative management. Primary outcome was defined as: residual or recurrent CSDH >10 mm, reoperation, or major neurological events at 180 days. It occurred in 16% versus 36% (OR 0.36; 95% CI 0.20–0.66; *p* = 0.001). The benefit was particularly significant in patients in whom standard treatment was conservative management (failure reached 56% in the control group versus 19% in the embolization group). Pooling data across four RCTs (*n* = 1808), adjunctive MMAE was associated with significantly lower recurrence and progression (5.3% vs. 9.1%; RR 0.58; 95% CI 0.39–0.86; *p* = 0.03), without a significant difference in functional outcomes at 90 days [84].

To this day, no international consensus exists on optimal embolic agents, patient selection criteria, or procedural timing relative to surgery; however, we can draw three main clinical scenarios where MMAE should be discussed. As monotherapy, it represents a viable alternative for patients with moderate, mildly symptomatic CSDH who are poor surgical candidates due to advanced age, frailty, or anticoagulation [85]. As a post-surgical adjunct, level 1 evidence from EMBOLISE [82] and STEM [83] supports MMAE in reducing recurrence, particularly in high-risk patients. Finally, it has been reported as an effective rescue strategy in recurrent CSDH following failed primary treatment.

Finally, an emerging concept worth noting is the single-session combination of MMAE and surgical evacuation within a hybrid operating room [86]. Kim et al. recently reported a single-center retrospective experience of 51 patients comparing this approach to a staged strategy; clinical and radiographic outcomes were comparable between groups, and no significant gain in procedural efficiency was demonstrated. While technically feasible, this integrated approach remains at an early stage and warrants prospective evaluation before broader adoption [87].

## 8. Recurrence: A Biological Rather than Technical Failure?

Several factors associated with CSDH recurrence have been explored in the literature [88]. Additional predictors of postoperative recurrence in chronic subdural hematoma can be grouped into three interrelated domains.

Patient-related factors reflect systemic vulnerability, including impaired coagulation control, endothelial dysfunction, and reduced reparative capacity, which predispose to persistent microbleeding and inadequate membrane healing.

Hematoma-related factors capture biological and radiological instability, such as large volume, bilateral involvement, high density, or heterogeneous composition, all of which are markers of ongoing hemorrhagic activity and inflammatory remodeling within the hematoma cavity.

Treatment-related factors emphasize the impact of surgical strategy on local pathophysiology: insufficient evacuation, absence of drainage, or lack of adjunctive techniques may fail to interrupt the cycle of hyperfibrinolysis, vascular leakiness, and pathological angiogenesis.

Collectively, these factors highlight that CSDH recurrence is not merely a technical failure but the clinical expression of a persistent, self-sustaining biological process that requires individualized, mechanism-oriented management.

There have been numerous attempts to identify predictors of recurrence in chronic subdural hematoma (CSDH). Among the earliest and most widely recognized is the classification proposed by Nakaguchi et al. in 2001 [64], which correlates radiological features with postoperative recurrence risk. In their study of 106 patients with 126 CSDHs, hematomas were categorized based on internal architecture and radiological density into four types: homogeneous, laminar, separated, and trabecular. The authors observed that the separated CSDH subtype carried the highest recurrence rate, whereas the trabecular type exhibited the lowest. They also proposed a chronological evolution for CSDH, progressing from an initial homogeneous stage through laminar and separated forms, before reaching a final resolution phase represented by the trabecular type. Regarding anatomical distribution—classified into convexity, cranial base, and interhemispheric extensions—hematomas at the cranial base demonstrated a higher recurrence propensity, while convexity lesions posed the lowest risk. These findings have driven the development of contemporary classification systems that integrate the Nakaguchi framework with additional prognostic variables, including hematoma density and the degree of cortical atrophy. Notably, the model proposed by Lioi et al. [88] provides a more comprehensive framework for estimating the risk of CSDH recurrence.

At present, recurrence assessment relies predominantly on postoperative CT imaging. However, it is conceivable that this approach may soon be complemented—or even partially replaced—by the use of biomolecular markers, as already occurs in other fields [89,90,91] where such indicators can reliably predict the clinical course including the recurrence.

## 9. Conclusions

Chronic subdural hematoma may not represent a true ‘subdural’ entity and can be viewed as an intradural border cell-derived disorder, challenging traditional anatomical and pathophysiological concepts. Rather than a static blood collection, it represents a dynamic, self-perpetuating disease driven by inflammation, pathological angiogenesis, and hemostatic dysregulation.

Within this framework, persistence and recurrence reflect ongoing biological activity rather than purely technical failure of surgical evacuation. This paradigm shift supports the adoption of mechanism-oriented strategies, including dural-targeted interventions such as middle meningeal artery embolization and pharmacological modulation of angiogenesis and fibrinolysis.

Reframing CSDH as a biologically active intradural disease may help guide more effective, personalized, and multidisciplinary management approaches.

## Figures and Tables

**Table 1:** Overview of the pathophysiology of CSDH, highlighting the interplay between hyperfibrinolysis, consumption of coagulation factors, intrinsic pathway dysregulation, and activation of the kallikrein–kinin system.

Pathophysiol. Domain	Specific Mechanism	Key Mediators	Biological Consequence	Clinical/Rad. Correlate	Ref.
Hemostatic dysregulation	Local hyperfibrinolysis	tPA ↑, uPA ↑, plasmin ↑	Continuous fibrin degradation and failure of stable clot formation	Persistent liquefied hematoma; high recurrence	Katano et al., 2006; Park et al., 2011 [55,56]
	Consumption of coag. factors	Factors VII, XI, XII ↓; plasmin ↑	Blood progressively loses coagulation capacity	Poor surgical hemostasis; refractory bleeding	Otsuka et al., 2025 [60]
Dynamic clot remodeling	Explosive fibrinolysis phase	D-dimer ↑↑	Rapid clot dissolution with recurrent microbleeds	Heterogeneous CT pattern	Park et al., 2011 [56]
	Quiescent fibrinolytic phase	D-dimer ↓ but persistent	Slow liquefaction without resolution	Homogeneous CT pattern	Park et al., 2011 [56]
Intrinsic pathway derailment	FXII activation on basement membrane	FXII → FXIIa	Attempted intrinsic coagulation activation	Ineffective microbleed sealing	Jukema et al., 2016 [59]
	Plasmin- mediated FXII cleavage	FXIIa β- chain	Loss of surface- dependent coagulation; shift to kallikrein	Failure of coagulation amplification	Jukema et al., 2016 [59]
	Prekallikrein → kallikrein	FXIIa β- chain, kallikrein	Amplified kinin cascade activation	Increased vascular permeability	Fujisawa et al., 1995 [58].
Kallikrein–kinin system	Bradykinin release	Bradykinin ↑	Chronic endothelial leakiness and plasma exudation	Membrane edema; protein-rich fluid	Fujisawa et al., 1995; [58]. Sakthiyendran et al., 2025 [54]
Endothelial dysfunction	Junctional disruption	Bradykinin, cytokines	Formation of endothelial gaps	Persistent fluid leakage	Fujisawa et al., 1995 [58]
	Platelet degranulation	PDGF, VEGF ↑	Recruitment and activation of pericytes	Neovascularization of outer membrane	Yokota et al., 2024 [61]
Inflammation–angiogenesis coupling Fibrotic remodeling	Pathological angiogenesis	Immature microvessels	Fragile, leaky neovessels prone to rupture	Recurrent microhemorrhages	Yokota et al., 2024 [61]
	Chronic inflammation	Fibroblasts, ECM deposition	Hypertrophic fibrotic stroma	Thickened outer membrane	Sakthiyendran et al., 2025 [54]
Therapeutic implications	Fibrinolysis inhibition	TXA, PAI-1	Stabilization of clot	Reduced expansion/ recurrence	Sakthiyendran et al., 2025 [54]
	Vascular supply interruption	MMA embolization	Ischemia of pathological membrane	Hematoma resolution	Otsuka et al., 2025 [60]

↑ means “upregualtion”, ↓ is “downregulation”

## Data Availability

Not applicable.
